# The Role of Single Nucleotide Variants of *NOS1, NOS2,* and *NOS3* Genes in the Development of the Phenotype of Migraine and Arterial Hypertension

**DOI:** 10.3390/brainsci11060753

**Published:** 2021-06-07

**Authors:** Polina V. Moskaleva, Natalya A. Shnayder, Marina M. Petrova, Daria S. Kaskaeva, Oksana A. Gavrilyuk, Sergey V. Radostev, Natalia P. Garganeeva, Victoria B. Sharavii, Elena E. Vaiman, Regina F. Nasyrova

**Affiliations:** 1 V.F. Voino-Yasenetsky Krasnoyarsk State Medical University, 660022 Krasnoyarsk, Russia; stk99@yandex.ru (M.M.P.); dashakas.ru@mail.ru (D.S.K.); oksana.gavrilyuk@mail.ru (O.A.G.); ex_t@mail.ru (S.V.R.); 2 V.M. Bekhterev National Medical Research Center for Neurology and Psychiatry, 192019 Saint-Petersburg, Russia; vaimanelenadoc@gmail.com (E.E.V.); nreginaf77@gmail.com (R.F.N.); 3 Siberian State Medical University, 634050 Tomsk, Russia; garganeeva@gmail.com; 4 I.M. Sechenov First Moscow State Medical University, Sechenov University, 119991 Moscow, Russia; victoriasharavii@gmail.com

**Keywords:** migraine, arterial hypertension, comorbidity, nitric oxide, nitric oxide synthase, *NOS1*, *NOS2*, *NOS3*, genes, single nucleotide variants, single nucleotide polymorphisms

## Abstract

Migraine (M) and arterial hypertension (AH) are very common diseases. Today, there are a number of studies confirming and explaining their comorbidity. We searched PubMed, Springer, Scopus, Web of Science, Clinicalkeys, and Google Scholar databases for full-text English publications over the past 15 years using keywords and their combinations. The present review provides a synthesis of information about single nucleotide variants (SNVs) of *NOS1*, *NOS2*, and *NOS3* genes involved in the development of M and essential AH. The results of studies we have discussed in this review are contradictory, which might be due to different designs of the studies, small sample sizes in some of them, as well as different social and geographical environments. Despite a high prevalence of the M and AH phenotype, its genetic markers have not yet been sufficiently studied. Specifically, there are separate molecular genetic studies aimed to identify SNVs of *NOS1*, *NOS2*, and *NOS3* genes responsible for the development of M and those responsible for the development of AH. However, these SNVs have not been studied in patients with the phenotype of M and AH. In this review, we identify the SNVs that would be the most interesting to study in this aspect. Understanding the role of environmental factors and genetic predictors will contribute to a better diagnostics and exploration of new approaches to pathogenetic and disease-modifying treatment of the M and AH phenotype.

## 1. Introduction

Arterial hypertension (AH) is a common disease worldwide and is a key risk factor for fatal cardiovascular complications [1]. Migraine (M) is the second most common type of primary headache and the most common form of headache with a genetic predisposition [2]. Many studies support the hypothesis that patients with M have an increased risk of developing AH, while patients with AH seem to have an increased risk of M. This allows us to hypothesize about the existence of the M and AH phenotype. The relationship between M and AH is potentially of great pathophysiological and clinical interest and is being actively studied. The pathophysiological pattern is significantly different in the setting of chronic pain, in which the adaptive relationship between blood pressure and pain sensitivity changes significantly. The association between acute or chronic pain and cardiovascular changes has been confirmed by observations, and some of this circumstantial evidence is supported by experimental models and human studies [3]. AH and M may have common mechanisms such as endothelial dysfunction, lack of autonomic regulation of the cardiovascular system, and involvement of the renin-angiotensin system.

**Figure 1:**
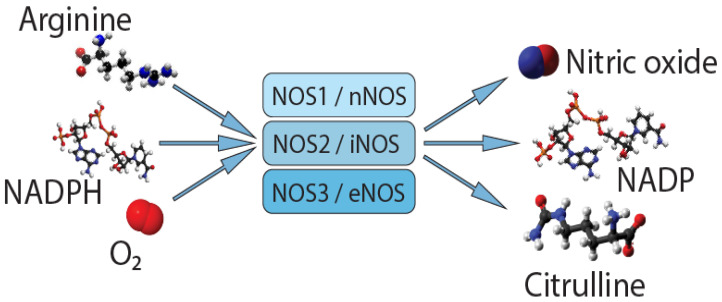
Synthesis of nitric oxide.

Nitric oxide (NO) is an important autocrine and paracrine signaling molecule that plays a crucial role in the regulation of the physiology and pathology of the cardiovascular system. NO is a very important molecule in the regulation of cerebral and extracerebral cranial blood flow and arterial diameter. Reduced bioavailability of NO in the endothelium is an important precursor to impaired vasodilation and hypertension. NO is also involved in nociceptive processing. NO synthase (NOS) is expressed in three isoforms (Figure 1): neuronal NOS (nNOS, NOS1), inducible NOS (iNOS, NOS2), and endothelial NOS (eNOS, NOS3) [4]. All NOS isoforms can catalyze the conversion of L-arginine to L-citrulline and NO. Active NOSs form a homodimer and convert the amino acid L-arginine to L-citrulline and NO. The NOS monomer contains a C-terminal reductase domain and an N-terminal oxygenase domain, which are linked by the calmodulin binding region (CaM). The N-terminal oxidase domain contains heme, tetrahydrobiopterin (BH4) cofactors, and an arginine substrate binding site. The oxidase domain is an active site for NO synthesis. NO production requires oxygen as an electron acceptor. NO diffuses freely across the plasma membrane and, therefore, is known to be transported to effector proteins in the same or adjacent cells and exerts its effects (for example, in smooth muscles, endothelial NO targets soluble guanylate cyclase, and sGC to ensure vasodilation) [5].

Single nucleotide variants (SNVs) of genes encoding NOSs can affect the level of their expression and/or activity in organs and tissues.

Increasingly more experimental evidence suggests that eNOS, iNOS, and nNOS have an important impact on cardiovascular function and pain [6,7]. Consequently, their combined effect on the M and AH phenotype in humans is of undoubted scientific and clinical interest. However, this phenotype requires a better treatment. In addition, there are many interesting associative studies of the role of *NOS1*, *NOS2*, and *NOS3* genes in the development of the M and AH phenotype.

Aim: analysis of associative studies of single-nucleotide variants of genes encoding NO-synthases in the migraine and arterial hypertension phenotype.

## 2. Materials and Methods

We searched PubMed, Springer, Scopus, Web of Science, Clinicalkeys, and Google Scholar databases for full-text English publications over the past 15 years using such keywords and their combinations as nitric oxide, nitric oxide synthase, NOS1, NOS2, NOS3, genes, single nucleotide variants, single nucleotide polymorphisms, comorbidity, arterial hypertension, and migraine. In addition, earlier publications of historical interest were included in the review.

We considered studies published from 2006 to 2021 and identified 52 publications devoted to the search for genetic predictors of the NO-synthase system in the development of migraine and AH. In our review, we summed up SNVs of *NOS1*, *NOS2*, and *NOS3* genes involved in the development of migraine and essential AH. Based on search criteria, only 38 of these publications were included in this review.

## 3. Results

Over the past 15 years, works aimed at finding associations of *NOS1*, *NOS2*, and *NOS3* genes with the development and course of headaches (H) have been carried out using the example of patients with M.

### 3.1. Migraine

#### 3.1.1. Gene *NOS1*

We found and analyzed three associative studies of the *NOS1* gene SNVs and the development of migraine, but there were no associations in any of them.

Alaşehirli et al. (2013) included 120 patients with M and 185 conditionally healthy volunteers from the Turkish population in their study. The results showed that the frequencies of alleles (*p* = 0.5257) and genotypes (*p* = 0.2841) of rs2682826 of the *NOS1* gene among patients with M did not statistically significantly differ from those of the control group [2].

Moreover, SNV rs2682826 was studied in the Japanese population. Ishii M. et al. (2014) compared the distribution of SNVs of three genes, including *NOS1* and *NOS3* (see M *NOS3*), in 47 patients with M and 22 patients with drug-induced H. The authors took into account the lack of association of this SNV with M in the Turkish population [2] and emphasized the need to continue work in this direction. They argued their choice of the following study design: during the M attack, the level of NO increases, at the same time it was shown that the synthesis of NO decreases during depression; and depressive disorders represent a comorbid state, which is more common with drug aggravation of H than attacks with M. However, the distribution of genotypes for SNV rs2682826 did not statistically significantly differ between groups (*p* = 0.254) [8].

García-Martín et al. (2015) continued to study the associations of SNVs of genes encoding NO synthases with the development of M in the Spanish population, with consideration of available biochemical, neuropathological, pharmacological, and experimental data. Accordingly, the poorly explored *NOS1* gene also fell into the area of their scientific interest. Taking into account the negative results of the work of Turkish and Japanese colleagues, García-Martín et al. included in their study other SNVs of the *NOS1* gene: rs693534 and rs7977109. However, the authors did not find significant differences in the distribution of genotypes and the frequency of alleles between the main (197 patients) and control (308 healthy Caucasians) groups [9].

#### 3.1.2. Gene *NOS2*

According to the results of studies of the *NOS2* gene SNVs, allelic associations and associations of genotypes were not found. However, several haplotypes were identified that increase the risk of M.

Specifically, Schurks et al. (2009) conducted a large-scale study of 77 SNVs of 52 candidate genes potentially associated with M, including the genes of the NO synthesis system, *OS2* and *NOS3* (see M *NOS3*), in the American population. A total of 25,713 women were examined, including 4705 patients with M and 21,008 women without a history of M. Among the *NOS2* gene SNVs, rs1137933 was considered. According to the results of genotyping, this SNV did not show a statistically significant association with a history of M [10].

De O.S. Mansur et al. (2012) studied two functional and, hypothetically, the most clinically significant SNVs of the *NOS2* gene (rs2779249 and rs2297518) in the Brazilian population, including 142 women without M and 200 women with M [11]. Another Brazilian study by Gonçalves et al. (2012) explored seven more SNVs: five SNVs of the *NOS3* gene (see M *NOS3*) and two SNVs of the *NOS2* gene, conducting a molecular genetic study of 99 apparently healthy women and 150 patients with M [12]. As a result, De O.S. Mansur et al. did not find associations of alleles or genotypes of the studied SNVs rs2779249 and rs2297518 with an increased risk of developing M, but showed that a haplotype with the simultaneous carriage of allele A was more common among patients suffering from M with aura than among the control group subjects (OR 2.65, 95% CI 1.34–5.22; *p* = 0.0027). [11]. Gonçalves et al. also did not find statistically significant differences in the frequency of the occurrence of alleles and genotypes of these *NOS2* SNVs between the control and main groups. At the same time, they further analyzed the gene–gene relationship and found a significant interaction when comparing the group of patients with M and the control group. The best interaction model included the rs743506 SNV of the *NOS3* gene and the rs2297518 SNV of the *NOS2* gene (*p* = 0.012). The combinations of genotypes with the highest risk of developing M included AA(rs743506)-AA(rs2297518), AA-GG, AG-GA, GG-GA, and GG-GG [12].

#### 3.1.3. Gene *NOS3*

The search for associations of the *NOS3* gene SNVs with M is carried out all over the world. However, the existing results are inconsistent.

Borroni et al. (2006) conducted a study in the Italian population, examining 156 patients with M (including 53 patients with aura) and 125 persons without H in general. The effect of functional SNV rs1799983 on the development of M was assessed. The authors showed that homozygous carriage of the major allele Asp298 increased the risk of developing M with aura in the studied population. According to the results, the AspAsp genotype of this SNV doubled the risk of developing M (OR 2.21, 95% CI 1.00–5.04; *p* = 0.05) and increased threefold the probability of having an aura (OR 3.02, 95% CI 1.21–7.51; *p* = 0.02) compared with the GluGlu + GluAsp genotypes. In addition, analysis of medical history and examination results showed no differences in clinical characteristics between groups. Therefore, according to the authors, this criterion is independent [13].

Toriello et al. (2008) continued the associative search in the Spanish population. The authors studied SNV rs1799983 as well as SNV rs1800779. The study included 337 patients with M (188 of them had aura) and 341 healthy volunteers as a control group. However, Spanish researchers were unable to confirm the results of their Italian colleagues. Thus, the frequencies of the alleles and genotypes of both SNVs did not differ between the groups (*p* > 0.01). None of the formed haplotypes influenced the development of M in the Spanish population. Toriello et al. explained these differences between their results and the results of Borroni et al. [13] by the fact that the Italian scientists did not carry out the correction for multiple hypothesis testing [14]. Nevertheless, taking into account the heterogeneity of the disease, it cannot be completely ruled out that the *NOS3* gene SNV is involved in the development of M, but with a lower risk.

In addition to their study of *NOS2* SNVs (see M *NOS2*), Schurks et al. (2009) explored three SNVs of the *NOS3* gene, including rs1799983, rs1800779, and rs3918226. When comparing patients with M with those of the control group, the authors did not find a difference in the frequency of occurrence of alleles and genotypes for all three SNVs. When dividing the patients into subgroups (1309 patients with M with aura and 1997 patients with M without aura) and from the subsequent intergroup comparison of each of the subgroups with the control group, an association of SNV rs3918226 with the development of M without aura was shown (OR 1.13, 95% CI 1.01–1.27; *p* = 0.04). However, after adjusting for multiple hypothesis testing, it also ceased to be statistically significant [10].

Gonçalves et al. studied five SNVs (rs2070744, rs3918226, variable number of tandem repeats of 27 pairs of nucleotides in intron 4 (VNTR 4 a/b), rs1799983, and rs743506) of the *NOS3* gene as possible markers of susceptibility to M in the Brazilian population. In 2011, they published the results of an associative study of SNV data with the development of M and aura in M. The sample consisted of 178 women with M (44 of them had aura) and 117 healthy controls. Allele frequencies did not differ between the study groups. When comparing genotypes, a statistically significant difference was revealed for SNV rs743506: the GA genotype was more common in the control group than among women with M (OR 0.47, 95% CI 0.29–0.78, *p* < 0.01). Then, 12 haplotypes were formed and studied, but no influence on the development of M was found. A relationship with the aura was shown: variants “C C a Glu G” and variants “C C b Glu G” were more common in women with M with aura than in women with M without aura (OR 30.71, 95% CI 1.61–586.4 and OR 17.26, 95% CI 1.94–153.4, respectively; both *p* < 0.0015625) [15]. A year later, the Brazilian research group published another paper, where they continued to study the five previously considered SNVs. Using an updated sample, Gonçalves et al. once again obtained the association of SNV rs743506 with M: the AA genotype was more common among patients with M and in the “migraine without aura” group than in the control group (*p* = 0.012 and *p* = 0.018, respectively). This work has already been mentioned by us above in relation to the SNV of the *NOS2* gene. Therefore, we recall that the authors also identified a number of combinations of genotypes with the highest risk of developing M for rs743506 of the *NOS3* gene and rs2297518 of the *NOS2* gene (see M *NOS2*) [12].

The only study found that also examined the VNTR 4 a/b polymorphism was conducted in the Turkish population [16]. It involved 105 patients with M and 97 healthy women. However, the authors did not find statistically significant differences between allele frequencies (*p* = 0.22) and genotypes *p* = 0.106) between patients with M and the control group, thus confirming the absence of an association between the development of M and this polymorphism [ibid].

In 2014 and 2015, two other research groups also carried out associative studies of the effect of the *NOS3* gene on the development of M in the Turkish population. However, despite the fact that the studies were carried out among people of the same nationality, the results were opposite. Eröz et al. (2014) studied variations of three SNVs (rs1799983, rs2070744, and rs3918226) in 176 patients with M and 123 healthy volunteers. The first two SNVs showed a statistically significant difference between patients with M and the control group (*p* < 0.0001). When comparing genotypes, the authors proved that, among patients with M, GT heterozygotes and TT homozygotes were much more common than among people without H (OR 3.027, 95% CI 1.830–5.008 and OR 3.221, 95% CI 1.223–8.484, respectively) in the study sample. Regarding SNV rs2070744, it was shown that heterozygous carriage of TC and homozygous CC were statistically significantly higher among patients with M relative to the control group (OR 2.843, 95% CI 1.681–4.808 and OR 3.729, 95% CI 1.784–7.792, respectively) in the study sample. The rs3918226 SNV showed no significant differences (*p* = 0.75) [17]. Güler et al. (2015) carried out a second study drawing on the example of the Turkish population [18]. The sample size was almost equal to that used by their colleagues [17] and amounted to 175 patients with M and 125 persons without H as a control group, while more SNVs were studied (rs743506, rs2070744, rs1799983, rs1800779, rs3918226, rs207468799, and rs148554851). However, none of these SNVs showed significant differences in the frequency of carriage of alleles or genotypes in the intergroup comparison (*p* > 0.05). Six haplotypes were formed among the patients. According to the results, none of the haplotypes was more common among patients with M compared with the control group [18].

In parallel with Turkish studies, a noteworthy study of genes *NOS1* and *NOS3* SNVs as risk factors for the development of medication overuse headaches (in patients with M) was carried out in the Japanese population. This work by Ishii et al. has already been described above (see M *NOS1*). SNV rs1799983 of the *NOS3* gene was studied. The distribution of genotypes was not statistically different between groups (*p* = 1.00) [8].

Zakerjafari et al. (2016) conducted a study in the Iranian population. The authors examined 120 persons, including 60 patients with M and 60 healthy subjects, to study SNV rs2070744. The frequency of carriage of the C allele significantly prevailed among patients with M compared with controls. When comparing genotypes, a statistically significant association was also revealed between this SNV and the development of M in the study population (*p* < 0.0001) [19].

Two large meta-analyzes were published in 2015 and in 2018.The first one reviewed associative studies of the rs1799983 SNV of the *NOS3* gene, and the second one explored the rs2070744 SNV of the same gene, as potential risk factors for the development of M. Chen et al. (2015) included six studies in their meta-analysis. The total number of participants was 1932 persons, including 1055 patients with M and 877 apparently healthy humans. As a result, no statistically significant association was found between the rs1799983 SNV and the risk of M in any of the studied genetic models among all participants. Analysis of subgroups by nationality showed that the T allele increased the risk of developing M among non-Caucasians (co-operative TT versus GG model: pooled OR 2.10, 95% CI 1.14–3.88) [20]. Dong et al. (2018) analyzed six studies, including a total of 1323 persons: 763 patients with M and 560 persons without H. Despite the fact that the authors studied another SNV (rs2070744), the results are largely similar to the meta-analysis of the rs1799983 SNV described above [20]. Thus, the absence of the effect of rs2070744 on the risk of developing M was demonstrated. However, analysis of different nationalities’ subgroups demonstrated that the CC genotype increased the risk of M compared with the TT + TC genotypes in Caucasian populations (OR 1.62, 95% CI 1.03–2.56, *p* = 0.04), with this association not observed among non-Caucasians with M (fixed-effects model; OR 0.88, 95% CI 0.51–1.53; *p* = 0.66) [21].

The results of the latest study in the Spanish population that we found on the subject of this review were published in 2020 by García-Martín et al. (2020), who observed 283 patients with M and 287 healthy volunteers. The authors used the results of a meta-analysis by Dong et al. [21] and studied SNV rs2070744 in representatives of the Caucasian race in Spain. However, the frequencies of alleles and genotypes did not differ significantly between the main and control groups (for the minor allele OR 0.91, CI 0.72–1.12, *p* = 0.418), although it was shown that homozygotes for the minor C allele were more common among patients with a burdened family history of M [22].

### 3.2. Arterial Hypertension

The association of SNVs genes encoding NOS with the risk of AH is being actively studied. Most of the works concern the *NOS3* gene SNV, while the *NOS2* gene SNV remains less well understood.

#### 3.2.1. Gene *NOS1*

There are very few studies on the association of *NOS1* gene SNVs with the risk of AH. However, we found one large study in the Swedish population, in which 58 SNVs of the NOS system genes were studied (see AH *NOS2* and *NOS3*), including the *NOS1* gene. A total of 3351 persons took part in the project, including 560 patients with coronary heart disease (CHD), 1158 patients with AH, and 1633 apparently healthy volunteers (control group). Levinsson et al. (2014) showed a stable statistically significant association of AH with the carriage of SNV rs3782218 (OR 0.81, 95% CI 0.67–0.97, *p* = 0.02; protective effect of the minor allele T), as well as with SNV rs7314935 (OR 2.15, 95% CI 1.06–4.37, *p* = 0.03; increased risk with the carriage of allele A) of the *NOS1* gene. Then, four haplotypes were formed. A protective effect of the GT haplotype (rs7314935-rs3782218) was shown against the other three haplotypes (OR 0.84, 95% CI 0.72–0.98, *p* = 0.03) [23].

#### 3.2.2. Gene *NOS2*

According to the results of Levinsson et al. [23] (see AH *NOS1*), of all studied SNVs of the *NOS2* gene, rs2255929 has a statistically significant effect on the risk of developing AH (OR 1.18, 95% CI 1.03–1.34, *p* = 0.02) [23].

Conen et al. (2009) analyzed 77 SNVs in 52 candidate genes, including *NOS2* and *NOS3* (see AH *NOS3*), in the American population. The authors evaluated the role of rs1137933 from the above-mentioned *NOS2* gene SNVs. The study involved 18738 women of the Caucasian race without AH at the time of inclusion. The development of AH was recorded in 5540 of these women after 9.8 years. The presence of gradual progression of AH was assessed within two years and was recorded in 47.4% of women, respectively. However, in carriers of alleles and genotypes of this SNV, no predisposition was shown either to the onset (OR 0.977, 95% CI 0.933–1.024, *p* = 0.33) or the progression of AH (OR 0.977, 95% CI 0.925–1.032, *p* = 0.41) [24].

In three subsequent associative studies of the *NOS2* gene with the development of AH, rs2297518 and rs2779249 and their haplotypes were studied. However, their results vary significantly. In particular, when conducting a study in the Brazilian population, Oliveira-Paula et al. (2013) examined 197 patients with AH and 113 volunteers with normal blood pressure. The analysis of genotypes showed that rs2297518 SNV increases the risk of developing AH (GG versus GA + AA: OR 2.05, 95% CI 1.16–3.75, *p* = 0.016), in contrast to the rs2779249 SNV. Then, eight haplotypes were formed (taking into account the microsatellite (CCTTT) n: S—short, <12). The SNVs haplotype was found six times more often among patients with AH than among patients with normal blood pressure (OR 6.07, 95% CI 1.57–29.27, *p* = 0.014) [25]. In a study by Nikkari et al. (2015), using the example of the Finnish population, the sample was larger and consisted of 320 hypertensive patients and 439 apparently healthy volunteers. The authors showed an association of the development of blood pressure with SNV rs2779249 (AA + AC increases the risk relative to CC: OR 1.47, 95% CI 1.08–2.01, *p* = 0.015), but not SNV rs2297518, in contrast to the results of colleagues from Brazil [25]. Haplotypes were also formed. As a result, a statistically significant effect on the risk of developing AH was shown by the haplotype with the simultaneous carriage of alleles A (OR 2.01, 95% CI 1.29–3.12, *p* = 0.002) [26]. Zhai et al. (2018), in turn, were also interested in the involvement of the *NOS2* gene SNVs in the pathogenesis of AH. They conducted a large study involving 1172 patients with AH and 1172 healthy individuals in the Chinese population. The frequencies of the alleles and genotypes were statistically significantly different between the groups in terms of SNV rs2297518 (*p* < 0.0001 in the frequency of alleles and *p* = 0.0006 in the frequency of genotypes) and in SNV rs2779249 (*p* < 0.0001 and *p* = 0.001, respectively). After the construction of logistic genetic models, OR also turned out to be significant in all models, except for the recessive-rs2297518. Specifically, the OR was 1.27 (95% CI 1.12–1.44) in the additive model, 1.31 (1.09–1.59) in the dominant model, and 1.68 (1.28–2.19) in the recessive model rs2779249; and 1.26 (1.06–1.50) in the additive model and 1.46 (1.13–1.89) in the dominant model rs2297518 [27].

#### 3.2.3. Gene *NOS3*

Endothelial NOS controls NO levels and ensures the normal functioning of vascular endothelial cells. As one of the key links in the development of AH is endothelial dysfunction, the main pool of associative molecular genetic studies is aimed at finding associations of the development of AH with SNVs in the *NOS3* gene.

As Conen et al. described (2009) [24] in the framework of the large American project “Research of the female genome” (see above AH *NOS2*), three *NOS3* SNVs were studied: rs1799983, rs1800779, and rs3918226. According to the results, only rs1799983 increased the risk of developing AH (OR 1.047, 95% CI 1.006–1.089, *p* = 0.03). However, after adjusting for multiple hypotheses testing, the association was no longer statistically significant. The associations of the studied alleles and genotypes of SNV with the progression of an increase in blood pressure were also insignificant [24]. Conen et al. (2008) published these results in another work, where they demonstrated the frequencies of the alleles and genotypes of the three studied SNVs in the study sample, and traced the influence of haplotype carriage on the development and progression of AH. However, there were no statistically significant associations with the progression of AH (*p* = 0.91) or the occurrence of AH (*p* = 0.1) [28].

In 2010, Kingah et al., within the framework of another American project “Atherosclerosis Risk in Community (ARIC)”, studied the association of rs1799983 with AH [29]. The sample was also large enough (15,792 persons), but more diverse, including both women and men, both Caucasians and African Americans. The reported results by Kingah et al. [29] differed from those of the previous studies [24,28]. The authors did not find statistically significant differences in the frequency of genotypes between the main and control groups, as well as depending on race (Caucasians: *p* = 0.8; African Americans: *p* = 0.5) [29].

Two groups of Chinese researchers, Li et al. [30] and Zhao et al. [31], analyzed the impact of SNVs on the development of AH among residents of the southwestern and northern regions of China. The first study involved 510 patients with AH and 510 persons with normal blood pressure as a control group. In the second study, the number of the study participants amounted to 503 and 490 persons, respectively. Li et al. (2011) evaluated the effect of SNVs rs2070744, rs1799983, and rs7830 on the development of AH. As a result, they revealed a statistically significant, after multiple comparisons, effect of SNV rs1799983 (OR 1.49, 95% CI 1.14–1.96, *p* = 0.003) and SNV rs7830 (OR 1.46, 95% CI 1.13–1.89, *p* = 0.004) on the development of AH in the Han-population in southwestern China. The analysis of haplotypes was also carried out, which showed the protective effect of the alleles G rs1799983 and G rs7830. Thus, of the eight haplotypes, TGG (rs2070744, rs1799983, and rs7830) was statistically significantly less common among patients with AH (OR 0.68, 95% CI 0.571–0.810, *p* = 1.49 × 10^−5^) [30]. Zhao et al. (2006), in turn, studied rs2070744 and rs1799983, as well as VNTR 4 a/b. However, unlike colleagues from the southern region of China [30], the authors did not find statistically significant differences in the frequency of occurrence of alleles and genotypes between the main and control groups. Eight haplotypes were formed, but, according to the results, none of them increased the risk of developing AH in the Han-population in the north of China [31].

The same three SNVs (rs2070744, rs1799983, and VNTR 4 a/b) were studied in parallel in the Brazilian and Singaporean populations. A study by Sandrim et al. (2006) involved 216 patients with AH and 111 apparently healthy Brazilians. Similar to the results of Zhao et al. [31], no statistically significant differences in the frequency of occurrence of alleles and genotypes were found. However, during the formation of haplotypes, a protective effect of the C-Glu-b haplotype (*p* < 0.00625) against AH was found as well as a provocative effect of the haplotype C-Asp-b (*p* < 0.00625) in the Brazilian population [32]. Moe et al. (2006), in turn, recruited 103 hypertensive patients and 104 healthy Singaporeans. The allele frequencies were statistically significantly different for VNTR 4 (*p* = 0.032), in contrast to rs2070744 (*p* = 0.207) and rs1799983 (*p* = 0.263). The bb VNTR 4 genotype was associated with the AH group (bb versus ba + aa + bc + ac: OR 1.8, 95% CI 0.9–3.4, *p* = 0.035), while no association of the rs2070744 and rs1799983 genotypes with AH was found (*p* = 0.419 and *p* = 0.227, respectively) [33].

Shankarishan et al. (2014) conducted a study in the Indian population. The study included 700 persons (350 patients with AH and 350 apparently healthy volunteers as a control group). The association of rs2070744, rs1799983, and VNTR 4 a/b with the risk of developing AH was studied. The results showed the following: genotype aa VNTR 4 (OR 6.81, 95% CI 2.29–20.25, *p* = 0.001) and TT genotype rs1799983 (OR 7.84, 95% CI 2.57–23.96, *p* < 0.001) were associated with AH. All three polymorphisms showed an association with the risk of AH when exposed to certain external environmental factors [34].

Another association study was conducted in the Sudanese population. Gamil et al. (2017) analyzed three SNVs: rs2070744, rs1799983, and VNTR 4 a/b. The study included 157 patients with AH and 85 healthy volunteers. The results were different from the data described above. Thus, only SNV rs2070744 showed an association with AH. The frequency of the C allele and the CC genotype was statistically significantly higher among patients with AH than among the control group (*p* = 0.03 and *p* = 0.02, respectively). Therefore, in the studied Sudanese population, SNV rs2070744 significantly increased the risk of developing AH (SS versus TS + TT: OR 2.14, 95% CI 1.23–3.74, *p* < 0.01) [35].

Moreover, we found a number of studies based on a different design and methodology that takes into account other parameters. In particular, in a genome-wide study of the association of the *NOS3* gene SNV with AH, Salvi et al. (2012) identified a locus (rs3918226) that increases the risk of developing AH (OR 1.54, 95% CI 1.37–1.73, *p* = 2.58 × 10^−13^) [36]. A year later, this group of scientists published the results of targeted sequencing, confirming that *NOS3* is a susceptibility gene for AH. The development of AH during the study in initially normotensive persons was statistically significantly associated with homozygosis of TT SNV rs3918226 (OR 2.04, 95% CI 1.24–3.37, *p* = 0.0054). This served as the basis for confirming the hypothesis that SNV rs3918226 is most closely associated with AH [37].

Levinsson et al. (2014, see AH *NOS1*) highlight the potential involvement of genes encoding NOS in the development of CHD and AH. According to their study, one SNV of the *NOS3* gene (rs3918226) increases the risk of developing AH (OR 1.32, 95% CI 1.01–1.72, *p* = 0.04) [23].

## 4. Discussion

Zicari et al. (2001) wrote: «*The involvement of nitric oxide (NO) in the pathophysiology of primary headaches was suggested by several authors during the last decade. Migraine, cluster headache, tension headache, and cervicogenic headache have been extensively studied on the basis of NO donor headache pain. Different mechanisms seem to be involved in the generation of pain in these clearly different clinical head pain disorders. NO could control all the mechanisms leading to head pain. In migraine NO is correlated with endothelial activation, in cluster headache with a brainstem unravelling of the on/off regulatory clocks, in cervicogenic headache with a cytokine-dependent pain, and in tension-type headache with a sensitization of pain pathways at the spinal/trigeminal level*» [38].

**Figure 2:**
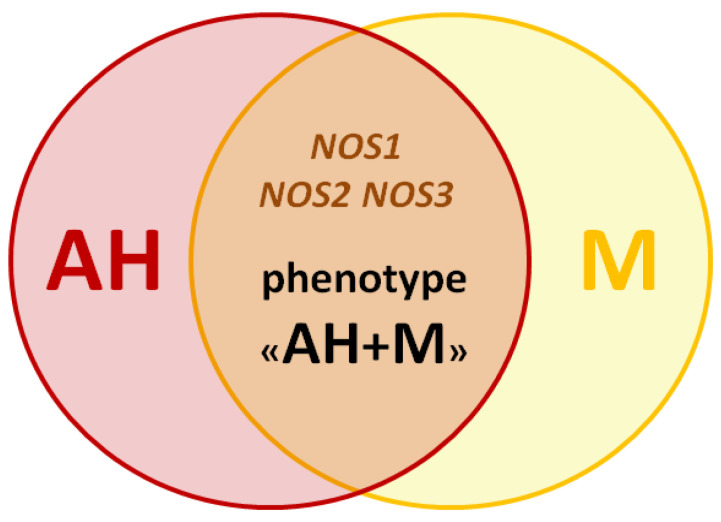
The scheme of the migraine and arterial hypertension phenotype.

Based on this review, we assume the presence of genetic predictors for the development of the clinical M and AH phenotype (Figure 2).

**Figure 3:**
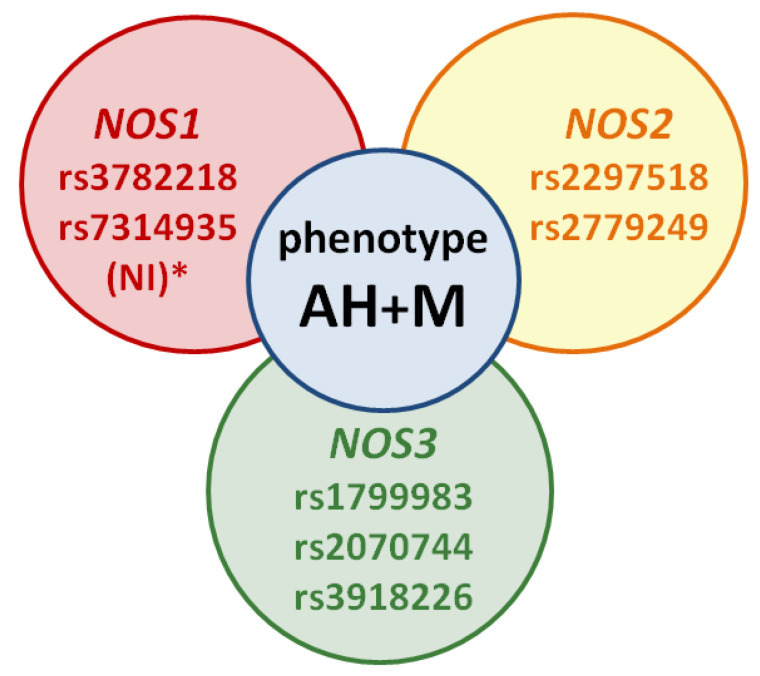
The scheme of the migraine and arterial hypertension phenotype: NI *—no or not enough information—SNVs’ studies on migraine versus AH are not available, but these SNVs may be potential markers of the phenotype in future studies.

The studied SNVs of genes *NOS1*, *NOS2*, and *NOS3*, in our opinion, are the most promising biomarkers for studying the genetic predisposition to the development of this phenotype. For the selection of such SNVs and their inclusion in your work, it is advisable to pay attention to the associative molecular genetic studies not only on the example of patients with AH and M (Table 1, Figure 3), but also with anxiety disorders and depression (according to the results of our research group’s previous literature review concerning genetic predictors of the NO-synthase system in the development of neuropsychiatric disorders [39]).

Of particular interest in the context of this review are works in which the *NOS1*, *NOS2*, and *NOS3* genes SNVs were considered as risk factors for several comorbid disorders, including AH and M or associated conditions. For example, MacClellan et al. (2009) studied the association of rs3918166 of the *NOS3* gene with the M and stroke phenotype [40], while Logan et al. (2005) studied the association of the same SNV with the glaucoma and M phenotype [41].

The importance of associative genetic studies of the phenotype of AN and M in the future is due to the similarity of some mechanisms of the development of these disorders, including the following:

- psychogenic mechanism (stress, anxiety, and psycho-emotional fatigue can provoke both the development of a M attack [42,43] and an episode of increased blood pressure [44,45]);
- vascular mechanism (vasodilation is one of the leading mechanisms for the development of M [46], and some antihypertensive drugs can cause excessive vasodilation of cerebral arteries and veins, leading to the development of migraine-like H [47]);
- biochemical mechanism (for example, NO-dependent vasospasm (impaired NO-dependent vasodilation) [46,48]).

## 5. Conclusions

Therefore, in our review, we synthesized information about SNVs of the *NOS1*, *NOS2*, and *NOS3* genes involved in the development of M and essential AH phenotype. The results of the studies we discussed in this review are inconsistent, which may be owing to different study designs; small sample sizes in some of them; and different racial, ethnic, social, and geographic characteristics. However, the contribution of genetic and environmental factors is still poorly understood, which forces researchers to actively study this problem. Understanding these mechanisms may facilitate the search for new approaches to pathogenetic and disease-modifying treatment of the M and AH phenotype as well as tension-type H and AH phenotype [49]. Inhibition of NO production, blockade of steps in the NO-cGMP pathway, or NO scavenging may be targets for new drugs for the treatment of AH and M. Indeed, selective inhibitors of n-NOS and i-NOS are already in early clinical development.

## Figures and Tables

**Table 1:** Association of genes *NOS1*, *NOS2*, and *NOS3* SNVs with the development of M and AH.

Gene	SNV	Migraine		Arterial Hypertension	
		Allele/Genotype	Haplotype	Allele/Genotype	Haplotype
*NOS1*	rs2682826	- [2,8]			
	rs3782218			+ [23]	+* [23]
	rs693534	- [9]			
	rs7314935			+ [23]	+* [23]
	rs7977109	- [9]			
*NOS2*	rs1137933	- [10]		- [24]	
	rs2255929			+ [23]	
	rs2779249	- [11,12]	+* [11]	+ [26,27] - [25]	+* [25,26]
	rs2297518	- [11,12]	+* [11,12]	+ [25,27] - [26]	+* [25,26]
*NOS3*	rs148554851	- [18]	-* [18]		
	rs1799983	+ [13,17,20] - [8,10,12,14,15,18]	-* [14,15,18]	+ [30,34] ± [24] - [28,29,31,32,33,35]	+* [30,32]-* [31]
	rs1800779	- [10,14,18]	-* [14,18]	- [24,28]	
	rs2070744	+ [17,19,21] - [12,15,18,22]	-* [15,18]	+ [35] - [30,31,32,33,34]	+* [30,32] -* [31]
	rs207468799	- [18]	-* [18]		
	rs3918226	± [10] - [12,15,17,18]	-* [15,18]	+ [23,36,37] - [24,28]	
	rs743506	+ [12,15] - [18]	+* [12] -* [15,18]		
	rs7830			+ [30]	+* [30]
	VNTR 4 a/b	- [12,15,16]	-* [15]	+ [33,34] - [31,32,35]	+* [32] -* [31]

Note: «+»—there is an association; «-»—there is no association; «+*» or «-*»—there is an association in the haplotype; «±»—the significant association disappeared after multiple comparisons.

## Data Availability

Not applicable.
